# Clinicopathological Study of 25 Cases of Diverticular Disease of the Appendix: Experience from Farwaniya Hospital

**DOI:** 10.1155/2013/404308

**Published:** 2013-10-02

**Authors:** Nabeel Al-Brahim, Ibrahim Al-Kandari, Musaad Munahai, Prem Sharma

**Affiliations:** ^1^Department of Pathology, Farwaniya Hospital, P.O. Box 3313, 22034 Salmiya, Kuwait; ^2^Department of Surgery, Farwaniya Hospital, 81004 Sabah Al-Naser, Kuwait; ^3^Faculty of Medicine, Kuwait University, P.O. Box 24923, 13110 Safat, Kuwait

## Abstract

*Background*. Diverticular disease of the appendix (DDA) is a rare disease and it has been shown to be associated with locoregional neoplasms. This study was conducted to characterize clinicopathological features and to investigate its association with appendiceal neoplasms. *Methods*. We searched the records of the Department of Pathology at Farwaniya Hospital for cases of diverticular disease of the appendix between 2003 and 2011. Histological slides and patient charts were reviewed for relevant information. Consecutive cases of acute appendicitis were selected as a control group. *Results*. We identified 25 cases of DDA, 24 of which occurred in men. Mean age of DDA patients was 35 ± 10.1 years and was significantly greater than that of appendicitis patients (*P* = 0.027). The mean temperature of cases (37.9°) was significantly higher (*P* = 0.012) than that of the controls (37.3°). The cases had lower white blood cell (WBC) counts compared to controls (13.6 versus 16.7, *P* = 0.04). Pathological diagnosis identified 4 cases of diverticulosis, 5 cases of diverticulitis, 6 cases of diverticulosis with acute appendicitis, and 10 cases of diverticulitis and appendicitis. None of the cases was associated with any type of neoplasm. *Conclusions*. DDA is a rare disease, and clinicians and radiologists should be aware of it. Male sex and adult age seem to be risk factors associated with DDA. The disease may not have any direct association with any neoplasm.

## 1. Introduction

Diverticular disease of the appendix (DDA) is a rare disease characterized by herniation or outpouching of the appendiceal mucosa through the muscular wall. Congenital DDA is rare, with a reported prevalence of 0.014%. It is equally frequent in both sexes and commonly presents as a single diverticulum [1]. Acquired DDA is also rare; it has been reported in 0.2% to 1.7% of all appendectomy specimens [2, 3]. In the medical literature, there are only few studies on DDA because the disease is extremely rare. Moreover, it is commonly overlooked by clinicians, radiologists, and surgeons because its clinical characterizations are limited, as is awareness of its complications. In relatively recent years, researchers have noticed an association between DDA and appendiceal neoplasms. Lamps et al. [4] reported a 25% association between appendiceal mucinous neoplasms and DDA. A study by Dupre et al. [5] revealed that 48% of appendectomy specimens with diverticulosis harbored appendiceal neoplasms including well-differentiated neuroendocrine tumors (carcinoid), mucinous adenoma, tubular adenoma, and adenocarcinoma. Furthermore, a recent study showed that 43.6% of the cases coincided with appendiceal neoplasm and regional colonic carcinoma [6]. Therefore, the authors considered DDA a marker of regional neoplasms.

This study was conducted at Farwaniya Hospital, a major general hospital in Kuwait, with the following objectives: (1) to further characterize the clinicopathological features of DDA in our population and compare them with the published data from different geographic areas; (2) to compare the cases of DDA with those of acute appendicitis; (3) to determine the association of DDA with appendiceal neoplasms.

## 2. Materials and Methods

We searched computer files (dated between 2003 and 2011) at the Department of Pathology at Farwaniya Hospital for the following terms: appendix, acute appendicitis, diverticular disease, diverticulosis, and diverticulitis. The cases of DDA in appendectomy specimens were included. Appendectomies associated with other procedures, such as right hemicolectomy and gynecological surgical procedures, were excluded because we used a simple computer software that allowed to enter the name of a single organ only. Therefore, appendectomies associated with other procedures were entered under the names of the procedure on other organs like colon and gynecological organs and thus difficult to identify. Moreover, the focus of this study was to select cases of DDA presenting as acute appendicitis so that they could be compared with actual cases of acute appendicitis (serving as controls). Histological slides were reviewed to confirm the diagnosis of DDA. In 14 cases, all appendices were submitted for histological examination. In the remaining cases (*n* = 11), we examined from 1 to 3 blocks of the appendix. Pathological diagnosis was defined as follows: (1) diverticulosis, defined as appendiceal mucosal herniation beyond the muscularis propria; (2) diverticulitis, which is characterized by the presence of acute inflammation around a diverticulum; (3) acute appendicitis, defined as the presence of transmural acute inflammation away from a diverticulum. Based on whether inflammation was present or absent and, if present, whether it affected a diverticulum or away from it, the pathological diagnosis was classified into four categories: (1) acute diverticulitis with normal appendix; (2) acute diverticulitis with acute appendicitis; (3) diverticulosis with acute appendicitis; (4) diverticulosis. Histological slides were also reviewed carefully for the presence of additional pathological findings—particularly neoplasms—and the number and location of the diverticula. Patient records were reviewed for demographic data, including age, sex, and nationality. Furthermore, clinical presentation, laboratory results, radiological imaging findings, and operative findings were recorded. Thirty-three consecutive patients who presented to hospital in 2010 with usual acute appendicitis were included as controls, and their records were reviewed for similar data.

### 2.1. Statistical Analysis

The data were entered and analyzed using the Statistical Package for the Social Sciences (SPSS) software, version 19.0 (IBM, Armouk, NY, USA). The normal distribution of age, duration of pain, temperature, white blood cell (WBC) count, and hospital stay were tested using Shapiro-Wilk test. Descriptive statistics were presented as means and standard deviations (SD) as well as medians and ranges, because these variables did not meet the assumptions of normal distribution. The chi-square or Fisher's exact test and Mann-Whitney *U* or Student's *t*-tests were used to compare cases and controls. A two-tailed *P* value of less than 0.05 was considered statistically significant.

The study was approved by a joint committee (Ministry of Health and Faculty of Medicine). 

## 3. Results

We identified 25 cases of DDA in the pathology files of Farwaniya Hospital. Twenty-four (96%) were men. Their ages ranged from 20 to 59 years, with a mean age of 35.0 ± 10.2 years. The mean age of DDA patients was significantly greater (*P* = 0.027) than that of the controls, 29.3 ± 8.69 years. The percentage of men was much higher among DDA group than that in the controls (96% versus 78%). Abdominal pain was found to be common among cases and controls, although the mean duration of pain was longer in patients with DDA. Nausea was slightly more frequent in patient with DDA cases, while vomiting and anorexia were more common among controls. The mean temperature was significantly higher in cases (37.9°) compared to controls (37.3°) (*P* = 0.012). The average duration of symptoms was 3 days with no significant associated diseases. The WBC count among cases was 13.6 ± 3.7, which was significantly lower (*P* = 0.041) than that of the control group, 16.7 ± 5.7. The mean duration of hospital stay was longer for patients with DDA than that for controls (4.7 days versus 4.1 days), although this was not a statistically significant difference (*P* = 0.077) (Table 1).

An ultrasound examination was performed for 10 patients. The findings were diagnostic of acute appendicitis in 4 patients, and no abnormalities were revealed in 3 patients. Fluid collection in the right iliac fossa and dilated bowel loops were observed in 2 patients, and the record for 1 showed fatty liver and no comment about the appendix. None of the cases were diagnosed with DDA based on imaging studies. We reviewed intraoperative findings with particular attention to the presence of perforation, diverticular disease, and pseudomyxoma peritonei. The records were available for 24 patients, 5 of whom had obvious evidence of perforation according to the surgeons. Pseudomyxoma peritonei was reported in 1 patient. DDA was not diagnosed intraoperatively in any of the patients. The remaining patients had an inflamed appendix. Three patients were reported to have an appendiceal mass.

### 3.1. Pathological Findings

**Figure 1:**
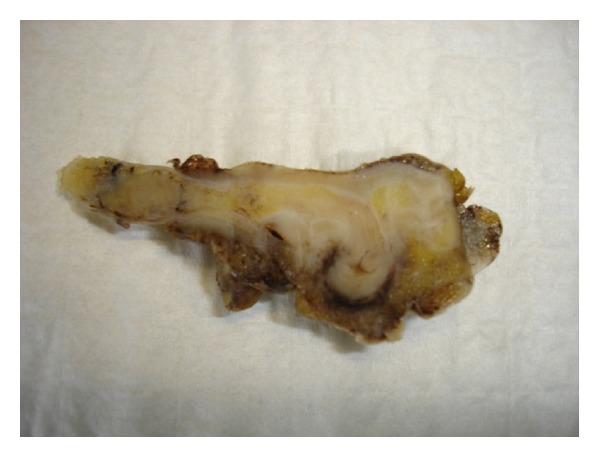
Gross picture of appendix with outpouching of the mucosa forming a diverticulum.

**Figure 2:**
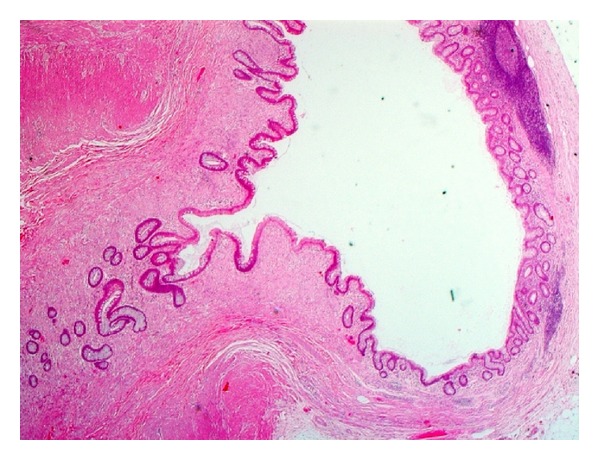
Histological picture of diverticulosis at the tip of the appendix (no inflammation around the diverticulum).

**Figure 3:**
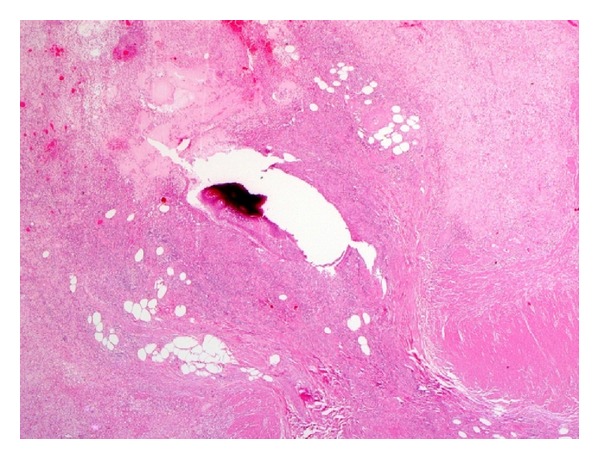
Histological picture of diverticulitis. Note the inflammatory cells around ruptured diverticulum.

**Figure 4:**
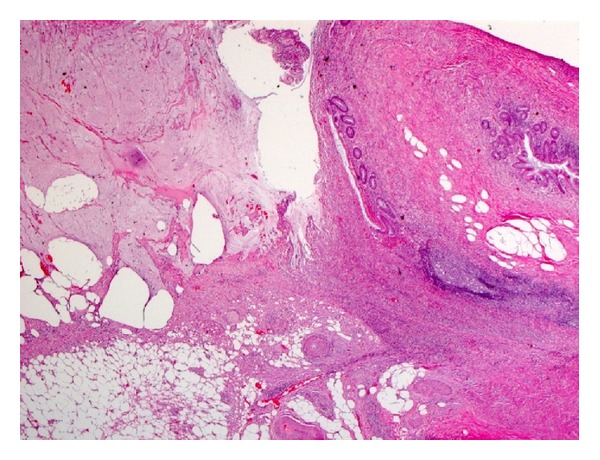
Histological picture of ruptured diverticulum. Note that localized mucinous spillage with epithelium on the serosa may resemble mucinous neoplasm.

Based on a histological examination of the appendectomy specimen, we differentiated 4 categories of disease in our patients: (1) diverticulosis, which indicates presence of a diverticulum without evidence of inflammation (*n* = 4); (2) diverticulitis, which indicates inflammation around the diverticulum (*n* = 5); (3) diverticular disease with acute appendicitis, which indicates inflammation in the appendix away from the diverticulum (*n* = 6); (4) diverticulitis and acute appendicitis, which indicates acute inflammation around the diverticulum and in the appendix away from the diverticulum (*n* = 10). The number of diverticula varied and ranged from 1 to 6. The tip of the appendix was the most common site of diverticula. There was no associated neoplasm in any of the cases (Figures 1, 2, 3, and 4).

## 4. Discussion

DDA is rare in appendectomy specimens. The mechanism of DDA was explained long ago by Stout [7]. The appendiceal mucosa herniates through penetrating vessels or the site of a previous perforation. Muscular contraction is also another contributing factor; muscular hypertrophy was seen in numerous patients with DDA. Furthermore, obstruction may play a role in the pathogenesis of DDA, as many cases with inspissated secretions and appendiceal neoplasms led to increased intraluminal pressure resulting from obstruction. DDA is commonly overlooked by clinicians and radiologists because it is so rare and clinicians are unfamiliar with its clinical presentations. Recently, this disease has attracted more attention because of studies reporting an association with appendiceal neoplasms and locoregional tumors [4–6].

In searching patient records at Farwaniya Hospital, which serves 1 million people, we identified only 25 cases of DDA occurring during a period of 9 years (2003–2011). This indicates just how rare the disease is. We found that the mean age of DDA patients (33.6 ± 8.33 years) was significantly older than that of the acute appendicitis group (29 ± 8.6 years). Lee et al. [8] obtained a similar result: the mean age of their study group (47.3 ± 3.0 years) was significantly (*P* = 0.001) greater than the mean age of their control group (31.8 ± 2.9 years). Another recent study also found that patients presenting with acute diverticulitis of the appendix were significantly older than those with acute appendicitis [9]. Various studies have found that DDA is more common in men. A study conducted by Lee and colleagues [8] found that 52% of patients were men, while two other studies [9, 10] reported that 66.6% and 83.3% of their respective series of cases were men. This can be explained by the fact that the majority of single expatriates in Kuwait are located in Farwaniya Governorate, the area served by the hospital. The clinical presentation of DDA in our series was characterized by higher temperature at presentation and lower WBC count, compared with acute appendicitis. Our series of cases in this study included 4 cases (16%) of DDA without inflammation (i.e., diverticulosis), and this explains the lower WBC count. However, there is no obvious explanation for the higher temperature in DDA patients than that in controls.

Imaging studies are important tools that help clinicians to diagnose patients and provide appropriate treatment. In this study, 10 patients (40%) underwent an ultrasound examination prior to surgical intervention, and none of them were diagnosed with DDA. This is likely because DDA is a rare disease and ultrasonographers are unfamiliar with radiologic findings. One study described the appearance of DDA on ultrasonography [11]. The morphology was characterized by an enlarged, swollen appendix and a small finger-like lateral projection. However, the literature shows that computed tomography (CT) is a more useful tool for detecting DDA if the practitioner is familiar with the disease. In a study by Lee et al. [8], appendiceal diverticulitis was included in preoperative diagnoses of 15% of the patients. However, with a greater awareness of DDA, the radiologist was able to recognize the disease in 80% of the cases. The CT finding that helped identify appendiceal diverticulitis was the presence of a small round cystic outpouching at the distal appendix with contrast enhancement of the cyst wall.

Pathological classification of DDA, as stated earlier, includes 4 categories: (1) acute diverticulitis with a normal appendix, (2) acute diverticulitis with acute appendicitis, (3) diverticulosis with acute appendicitis, and (4) diverticulosis. The literature shows that the first category is the most common while in our study it was the second category. These findings can be attributed to late presentation of the patients, who live in an area associated with low socioeconomic status and are usually late in seeking medical advice.

Recently, DDA has attracted more attention from clinicians and researchers because an association with locoregional neoplasm has been found. Lamps and colleagues [4] reviewed cases of low-grade mucinous neoplasm, diverticulosis, and adenoma in appendectomy specimens. They identified 32 cases of mucinous neoplasm, of which 8 (25%) were associated with a diverticulum. Therefore, the authors calculated the probability of observed rate of occurrence of mucinous neoplasm and diverticulosis which was 42%. This is higher than the observed rates of each separate condition reported in the literature, which ranged from 1% to 2%. They explained this finding by the fact that the dysplastic epithelium produced mucin, which increased the intraluminal pressure, resulting in mucosal herniation. Another study, by Dupre et al. [5], found that diverticulosis was present in 23 patients (1.7%) of a series of 1361 cases, of which 11 harbored appendiceal neoplasm, including 5 well-differentiated neuroendocrine tumors (carcinoid), 3 mucinous adenomas, 2 mucinous adenocarcinomas, and 1 tubular adenoma. Therefore, they calculated that 45% of the cases of mucinous lesions, either invasive or noninvasive, were associated with diverticular disease. Interestingly, DDA was associated with 5 cases of well-differentiated neuroendocrine tumor. This type of tumor commonly occurs at the tip of the appendix. However, the authors of the previously mentioned study reviewed all cases of carcinoid tumor of the appendix and observed that tumors associated with diverticular disease were located in the mid or proximal appendix rather than the tip. This indicates that DDA resulted from obstruction and increased intraluminal pressure.

There is a difference between the data published in the literature and the data showed herein regarding the association of DDA with appendiceal tumors, particularly mucinous neoplasm. Different explanations for this discrepancy can be extrapolated. For example, sampling of the appendix may play a role in this difference as the more sections from the appendix are taken for histological examination, the more chance of identifying these neoplasms. In addition, understanding the histological morphology of mucinous neoplasm may help in understanding this contradictory finding. These tumors commonly have a flat or villous epithelium with a flat lymphoid follicle. The cells are filled with mucin and the nuclei are columnar with low-grade dysplasia [12]. With such subtle diagnostic features, the interobserver variability of pathologists is high. The distinction of these tumors from retention cysts or reactive atypia that is seen in the presence of inflammation may be difficult. Therefore, these low-grade features may be misinterpreted as low-grade mucinous neoplasm. Another explanation is that ruptured appendiceal diverticula with periappendiceal mucin spillage, particularly in some cases with localized pseudomyxoma peritonei, can mimic low-grade appendiceal neoplasm (as shown in Figure 4). Hsu et al. [13] published a series of consultation cases of ruptured appendiceal diverticula that were misdiagnosed as low-grade mucinous neoplasm or flagged as suspicious by the original pathologist. We conclude that reactive nuclear atypia seen in diverticulitis, ruptured diverticulum, and localized mucin spillage may be overinterpreted as low-grade mucinous neoplasm. 

In conclusion, diverticular disease of the appendix is a rare disease; male sex and adult age are risk factors. Clinicians and radiologists are still widely unaware of its clinical features. The literature shows that CT is the best imaging modality for diagnosis. Our study did not show any association of DDA with appendiceal neoplasm. Therefore, pathologists should be alert not to overdiagnose any reactive atypia or ruptured diverticulum as low-grade mucinous neoplasm.

## Figures and Tables

**Table 1:** Summary of clinical presentation of the cases and control.

Demographic and clinical presentation	Diverticular disease of appendix (*n* = 25)	Acute appendicitis (*n* = 33)	*P *
*Sex *			
Male	24 (96%)	26 (78.2%)	0.121
Female	1 (4%)	7 (21.2%)	
*Age, years *			
Mean ± SD	35.0 ± 8.33	29.3 ± 8.69	0.027
Median (range)	34 (20–56)	27 (12–51)	
*Ethnic group *			
Arabs	14 (56%)	16 (48.5%)	0.571
Asian	11 (44%)	17 (51.5%)	
*Pain *			
Present	25 (100%)	33 (100%)	—
Absent	0	0	
*Duration of pain, days *			
Mean ± SD	2.89 ± 2.4	2.06 ± 2.05	0.108
Median (range)	2 (0.25–10)	2 (0–12)	
*Nausea *			
Present	12 (48.0%)	13 (39.4%)	0.512
Absent	13 (62.0%)	20 (60.6%)	
*Vomiting *			
Present	9 (36.0%)	20 (60.6%)	0.063
Absent	16 (64.0%)	13 (39.4%)	
*Temperature *			
Mean ± SD	37.9 ± 0.72	32.8 ± 0.73	0.012
Median (range)	37.9 (36.6–39.6)	37.2 (36.3–39.4)	
*Anorexia *			
Present	7 (28.0%)	21 (63.6%)	0.007
Absent	18 (72.0%)	12 (36.4%)	
*WBC *			
Mean ± SD	13.6 ± 3.7	16.7 ± 5.75	0.041
Median (range)	14.2 (4.5–20.8)	16.2 (6.8–36.5)	
*Hospital stay *			
Mean ± SD	4.72 ± 1.67	4.09 ± 2.04	0.077
Median	4 (3–10)	4.0 (1–10)	
